# A Case of Recurrent Painful Ophthalmoplegia Following COVID‐19: Evolution From Optic Neuritis to Tolosa–Hunt Syndrome

**DOI:** 10.1002/ccr3.71686

**Published:** 2025-12-17

**Authors:** Sanjiv Poudel, Sumee Pradhan, Rimi Pradhan, Adarsha Mahaseth, Bidhya Pandey, Amrit K C

**Affiliations:** ^1^ Lakecity Hospital & Critical Care Pvt. Ltd Pokhara Nepal; ^2^ Narayani Samudayik Hospital (NPI‐NSH) Bharatpur Nepal; ^3^ Dev Hospital Pvt. Ltd Bharatpur Nepal; ^4^ Nepalese Army Institute of Health Sciences (NAIHS) Kathmandu Nepal; ^5^ Mount Sinai Hospital Chicago Illinois USA; ^6^ Dr. Iwamura Memorial Hospital Bhaktapur Nepal

**Keywords:** cavernous sinus inflammation, cranial neuropathy, granulomatous inflammation, neuro‐ophthalmology, optic neuritis, orbital apex syndrome, painful ophthalmoplegia, post–COVID neurological syndrome, steroid‐responsive syndrome, Tolosa–Hunt syndrome

## Abstract

Tolosa–Hunt syndrome could be considered in patients with painful ophthalmoplegia following COVID‐19 infection, but caution should be exercised in attributing causality. Careful exclusion of alternative diagnoses is essential. Corticosteroids usually produce quick clinical relief. Ultimately, any association with COVID‐19 is regarded as speculative and unproven.

## 1 Introduction

Severe acute respiratory syndrome coronavirus 2 (SARS‐CoV‐2) is known to involve multiple organ systems, including the ocular and neuro‐ophthalmic pathways [1]. Ocular manifestations of coronavirus disease 2019 (COVID‐19) range from conjunctivitis [2] to vascular events such as central retinal artery occlusion (CRAO) and retinal vein occlusion (RVO) following infection [3]. Additionally, neuro‐ophthalmic complications including optic neuritis and cranial nerve palsies (e.g., oculomotor palsy) have been reported in association with COVID‐19 [4, 5]. Although these disorders remain relatively rare, case reports indicate that optic neuritis and other neuro‐ophthalmic sequelae typically respond well to corticosteroid therapy [6].

Tolosa–Hunt syndrome (THS) is a rare, idiopathic, granulomatous inflammation of the cavernous sinus and superior orbital fissure, causing severe unilateral periorbital headache and painful ophthalmoplegia [7, 8]. It is uncommon and according to the most recent epidemiological data, THS has an estimated annual incidence of approximately one case per million population [9]. THS is characterized clinically by ipsilateral paresis of one or more ocular motor nerves (III, IV, and/or VI) and headache, often with imaging evidence of inflammation in the cavernous sinus or orbital apex [8, 10]. High‐dose glucocorticoids typically produce a rapid resolution of pain and improvement in nerve palsies [10, 11].

We report a patient with recurrent episodes of painful ophthalmoplegia temporally related to COVID‐19 infection. The first episode, initially diagnosed as optic neuritis, may have represented early orbital apex involvement by the same inflammatory process. The second episode fulfilled clinical and radiographic criteria for THS. The temporal sequence is intriguing but any causal link with SARS‐CoV‐2 remains conjectural.

## 2 Case Presentation

A 50‐year‐old Nepali man with well‐controlled hypertension (on amlodipine) and a remote history of treated pulmonary tuberculosis presented to a tertiary care center in Kathmandu, Nepal, with acute‐onset left periorbital pain and visual loss. Reverse transcriptase‐polymerase chain reaction (RT‐PCR) had confirmed SARS‐CoV‐2 infection 4 weeks earlier, for which the patient required prolonged ICU stay, mechanical ventilation, and high‐dose dexamethasone. After discharge, the patient recovered at home on a tapering steroid course over 4 weeks. The patient denied diabetes, HIV risk factors, recent vaccination, ocular trauma, or other viral illnesses.

Two weeks after completing prednisone taper, the patient developed acute blurring of vision in the left eye, associated with periorbital pain exacerbated by eye movement. There was no trauma, vaccination, systemic neurological symptoms, or prior similar episodes. On examination, visual acuity was counting fingers at 2 m in the left eye and 20/20 in the right, with a left‐sided relative afferent pupillary defect. Fundoscopy revealed mild left optic disc edema; ocular motility was full without ptosis or diplopia. A contrast‐enhanced MRI of the brain and orbits showed enhancement of the left optic nerve sheath extending to the orbital apex, consistent with optic neuritis; there was no cavernous sinus inflammation or intracranial demyelinating lesions. Given resource constraints, CSF studies and advanced serologic testing (anti‐MOG, aquaporin‐4 antibodies) were not performed, but basic laboratory tests including CBC, ESR, CRP, and fasting glucose were all normal. A presumptive diagnosis of post‐viral optic neuritis was made, and treatment with IV methylprednisolone (1 g/day for 3 days) was started, followed by an oral prednisone taper. The patient's vision improved to 20/60 over 2 weeks.

Six weeks later, the patient returned with severe constant retro‐orbital headache and horizontal binocular diplopia that began 1 day after the pain. On exam, visual acuity was 20/20 bilaterally, pupils were equal and reactive, and fundoscopy was normal. The patient had partial left ptosis, limitation of adduction and abduction (III and VI nerve palsies), and decreased sensation in the V1 distribution; findings consistent with cavernous sinus syndrome.

Repeat contrast‐enhanced MRI revealed abnormal enhancement and thickening of the left cavernous sinus extending into the superior orbital fissure and orbital apex, with no mass lesion or carotid involvement. These findings are radiologically consistent with THS. Magnetic resonance angiography ruled out aneurysm, fistula, or vascular mal formation. A comprehensive investigative work‐up was undertaken to exclude alternative etiologies. Routine blood tests—including complete blood count, erythrocyte sedimentation rate, C‐reactive protein, fasting blood glucose, and liver and renal function panels—were all within normal limits, and thyroid function tests likewise showed no abnormalities. Autoimmune screening with antinuclear antibody (ANA) and anti–neutrophil cytoplasmic antibody (ANCA) tests returned negative, and serologic testing for syphilis (VDRL) and HIV was also negative.

The QuantiFERON‐TB Gold assay was positive, which reflects prior exposure to tuberculosis. However, chest radiography and computed tomography revealed only old fibrotic scars without any signs of active pulmonary disease. Cerebrospinal fluid analysis demonstrated a normal opening pressure; the fluid was acellular, with normal glucose and protein concentrations, and cultures as well as PCR assays for bacteria, fungi, and *Mycobacterium tuberculosis* were all negative. Serum angiotensin‐converting enzyme (ACE) levels were within the reference range, making neurosarcoidosis unlikely.

Given regional resource constraints, advanced or non‐endemic investigations, such as Lyme serology, anti‐SSA/SSB antibodies, viral serologies for CMV, HSV, and West Nile virus, as well as optical coherence tomography and fluorescein angiography were not performed. Nevertheless, the combination of thorough clinical assessment and characteristic radiologic findings was considered sufficient to establish the diagnosis.

## 3 Differential Diagnosis

Infectious cavernous sinusitis (bacterial or fungal) was unlikely given afebrility, normal WBC, and negative cultures. Granulomatous infections (TB, syphilis) were excluded by imaging and serologies. Neoplasm (meningioma, lymphoma, metastasis) was ruled out by MRI appearance and lack of systemic signs or biopsy. Carotid‐cavernous fistula/aneurysm was excluded by normal MRA. Vasculitides (giant cell arteritis, GPA) were unlikely with normal ESR/CRP and negative ANCA. Neurosarcoidosis or IgG4‐related disease lacked supporting parenchymal lesions, elevated ACE, or chest findings. Microvascular palsies (diabetic, hypertensive) and thyroid eye disease were inconsistent with the intense pain and cranial nerve involvement. Myasthenia gravis and ophthalmoplegic migraine were excluded by clinical features and demographic profile. After exclusion of these, THS remained the leading diagnosis.

## 4 Management

The patient received high‐dose corticosteroids: IV methylprednisolone 1 g daily for 5 days, followed by oral prednisone 1 mg/kg daily tapered over 8 weeks, with proton‐pump inhibitor and calcium/vitamin D supplementation. Pain resolved within 48 h; ocular motility normalized by 2 weeks. To reduce relapse risk, mycophenolate mofetil was initiated at 500 mg twice daily, titrated to 1 g twice daily, with regular hematologic and biochemical monitoring and trimethoprim‐sulfamethoxazole prophylaxis. At 6‐month follow‐up, he remained asymptomatic with full extraocular movements.

## 5 Discussion

THS is an idiopathic, non‐specific granulomatous inflammation of the cavernous sinus and superior orbital fissure. Patients typically present with unilateral orbital pain and paresis of cranial nerves III, IV, and/or V. These nerves course through the cavernous sinus and orbital apex, so involvement often extends into the superior orbital fissure or apex. Our patient's presentation with ipsilateral headache and ophthalmoplegia affecting multiple ocular motor nerves fulfilled the revised International Classification of Headache Disorders criteria for THS [12]. MRI usually demonstrates an enlarged or enhancing cavernous sinus wall, as seen in this case [13, 14]. By definition, THS is a diagnosis of exclusion after infectious, neoplastic, and other inflammatory causes have been ruled out [15].

Our case is notable for two temporally distinct episodes. The first event was diagnosed as typical post‐viral optic neuritis; an isolated optic nerve inflammation. The second event was classic THS. On reflection, it is possible that the initial optic neuritis represented early orbital apex involvement of the same inflammatory process. THS can involve the orbital apex and compress the optic nerve; in some reports THS and orbital apex syndromes overlap. If the first episode was indeed a mild form of the same granulomatous inflammation, it would explain the later recurrence. Alternatively, it may represent two separate phenomena (optic neuritis followed by THS). Either way, the progression highlights that painful ophthalmic events after COVID‐19 can evolve over time and should prompt reconsideration of prior diagnoses.

Similar cases have been reported in the literature. Several neuro‐ophthalmic complications, including THS and other orbital inflammatory conditions, have been reported both after SARS‐CoV‐2 infection and following COVID‐19 vaccination. For instance, the first pediatric case of THS post‐COVID‐19 was described in a previously healthy 12‐year‐old girl who developed painful ophthalmoplegia with MRI evidence of cavernous sinus inflammation 1 week after confirmed SARS‐CoV‐2 infection, responding rapidly to high‐dose corticosteroids [16]. A 65‐year‐old diabetic woman developed acute complete external ophthalmoplegia in association with SARS‐CoV‐2 infection, with spontaneous partial recovery [17]. A similarly rare presentation involved THS complicated by hemorrhagic encephalitis in an adult with COVID‐19, highlighting the broader spectrum of granulomatous neuro‐inflammation associated with the virus [18]. Beyond THS, orbital myositis has been documented in adults shortly after SARS‐CoV‐2 infection, where patients presented with periorbital edema, proptosis, and gaze limitation, with MRI confirmation of extraocular muscle inflammation that resolved with systemic steroids [19]. On the vaccination front, Ammari et al. reported a rare case of THS in a 64‐year‐old patient who developed recurrent horizontal diplopia and ipsilateral orbital pain 2 weeks after COVID‐19 vaccination, was diagnosed after exclusion of other causes, improved with corticosteroids, and whose presentation suggests an autoimmune response linking THS to vaccination [15]. Similarly, Chuang et al. reported a case of THS arising 5 days after administration of an mRNA COVID‐19 vaccine (mRNA‐1273), characterized by cavernous sinus enhancement on MRI and rapid symptomatic relief with corticosteroid therapy [20]. Moreover, diffuse orbital inflammation presenting as periorbital swelling, pain, and diplopia has been observed within days of mRNA vaccination, with imaging confirming capsulitis‐like changes and favorable response to steroids [21]. Ang et al. described three cases of orbital inflammation, with one THS occurring 14 days after a booster dose and two instances of orbital myositis presenting 1–10 days after initial or subsequent Pfizer‐BioNTech COVID‐19 vaccinations. The cases presented with characteristic MRI findings, unremarkable autoimmune workups, and complete resolution either with systemic or local corticosteroids (or spontaneously), thereby identifying orbital inflammation as a rare adverse effect of vaccination [22]. These reports include isolated THS as well as orbital myositis and dacryoadenitis. However, such case reports are anecdotal and cannot establish causality. The association remains speculative. It is biologically plausible that a robust immune response to the virus (or vaccine) could trigger an autoimmune orbital inflammation, but no definitive mechanism has been proven. We emphasize that in our patient the temporal link is intriguing but not definitive; the patient's THS might well have occurred independently of the patient's COVID‐19 infection.

The rapid response to corticosteroids was consistent with reported THS cases [15, 18, 19, 20]. High‐dose steroids are both diagnostic and therapeutic, as dramatic pain relief supports the inflammatory hypothesis. We did not administer any additional immunosuppression, and the patient has remained symptom‐free off therapy, although relapses are described.

## 6 Limitations

This case has several limitations. It describes the experience of one individual, so the clinical observations may not apply broadly. There is no tissue diagnosis, since a biopsy of the cavernous sinus was not pursued and the conclusion relied on clinical features and radiologic clues. Because of this, an uncommon tumor or a slow‐growing inflammatory condition that resembles THS on imaging cannot be entirely dismissed. Furthermore, several diagnostic studies including cerebrospinal fluid assays for MOG and aquaporin‐4 antibodies were not available in our setting, which means that antibody‐associated optic neuritis and related inflammatory disorders cannot be fully excluded. The temporal link with COVID‐19 is descriptive rather than proved, since no biological marker or population‐level data exists to establish a direct relationship. Additional reports and structured investigations would be needed to clarify whether SARS‐CoV‐2 can precipitate THS or similar orbital inflammatory events.

## 7 Conclusion

We describe a patient with recurrent painful ophthalmoplegia initially diagnosed as optic neuritis and later as THS, in close temporal relation to COVID‐19. This case highlights that atypical orbital inflammations can occur after viral infections, but clinicians should be cautious in attributing causality. THS should be considered in patients with painful ocular motor palsies, and prompt steroid therapy can lead to rapid recovery. Given the single‐case nature and the lack of definitive evidence, any association between COVID‐19 and THS remains uncertain. Further study is needed to clarify any link between SARS‐CoV‐2 and cavernous sinus inflammation.

## Author Contributions

**Sanjiv Poudel:** conceptualization, project administration, visualization, writing – original draft, writing – review and editing. **Sumee Pradhan:** data curation, validation, writing – original draft, writing – review and editing. **Rimi Pradhan:** data curation, validation, writing – original draft, writing – review and editing. **Adarsha Mahaseth:** data curation, investigation, methodology, resources, supervision, writing – review and editing. **Bidhya Pandey:** data curation, methodology, writing – review and editing. **Amrit K C:** methodology, validation, writing – review and editing.

## Funding

The authors have nothing to report.

## Ethics Statement

The authors have nothing to report.

## Consent

Written informed consent was obtained from the patient for publication of this case report and accompanying images. A copy of the consent form is retained by the authors and is available for review upon request.

## Conflicts of Interest

The authors declare no conflicts of interest.

## Data Availability

Data sharing is not applicable to this article as no datasets were generated or analyzed during the current study.
